# Conjugation to the sigma-2 ligand SV119 overcomes uptake blockade and converts dm-Erastin into a potent pancreatic cancer therapeutic

**DOI:** 10.18632/oncotarget.9551

**Published:** 2016-05-22

**Authors:** Kerri A. Ohman, Yassar M. Hashim, Suwanna Vangveravong, Timothy M. Nywening, Darren R. Cullinan, S. Peter Goedegebuure, Jingxia Liu, Brian A. Van Tine, Herve Tiriac, David A. Tuveson, David G. DeNardo, Dirk Spitzer, Robert H. Mach, William G. Hawkins

**Affiliations:** ^1^ Department of Surgery, Washington University School of Medicine, St. Louis, MO, USA; ^2^ Department of Radiology, Washington University School of Medicine, St. Louis, MO, USA; ^3^ Alvin J. Siteman Cancer Center, Barnes-Jewish Hospital, and Washington University School of Medicine, St. Louis, MO, USA; ^4^ Division of Public Health Sciences, Section of Oncologic Biostatistics, Washington University School of Medicine, St. Louis, MO, USA; ^5^ Division of Medical Oncology, Washington University School of Medicine, St. Louis, MO, USA; ^6^ Cold Spring Harbor Laboratory, New York, NY, USA; ^7^ Department of Pathology and Immunology, Washington University School of Medicine, St. Louis, MO, USA; ^8^ Department of Radiology, University of Pennsylvania, Philadelphia, PA, USA

**Keywords:** sigma-2 receptors, erastin, pancreatic cancer, targeted therapy, selective delivery

## Abstract

Cancer-selective drug delivery is an important concept in improving treatment while minimizing off-site toxicities, and sigma-2 receptors, which are overexpressed in solid tumors, represent attractive pharmacologic targets. Select sigma-2 ligands have been shown to be rapidly internalized selectively into cancer cells while retaining the capacity to deliver small molecules as drug cargoes. We utilized the sigma-2-based drug delivery concept to convert Erastin, a clinically underperforming drug, into a potent pancreatic cancer therapeutic. The Erastin derivative des-methyl Erastin (dm-Erastin) was chemically linked to sigma-2 ligand SV119 to create SW V-49. Conjugation increased the killing capacity of dm-Erastin by nearly 35-fold in vitro and reduced the size of established tumors and doubled the median survival in syngeneic and patient-derived xenograft models when compared to non-targeted dm-Erastin. Mechanistic analyses demonstrated that cell death was associated with robust reactive oxygen species production and could be efficiently antagonized with antioxidants. Mass spectrometry was employed to demonstrate selective uptake into pancreatic cancer cells. Thus, targeted delivery of dm-Erastin via conjugation to the sigma-2 ligand SV119 produced efficient tumor control and prolonged animal survival with minimal off-target toxicities, and SW V-49 represents a promising new therapeutic with the potential to advance the fight against pancreatic cancer.

## INTRODUCTION

Pancreatic cancer is a devastating disease with a 5-year survival of only 7% [1]. Surgical intervention remains the only potential cure, but only 15 to 20 percent of patients are diagnosed early enough to be amenable for an attempt at resection [2]. Even in this highly select group of patients, the prognosis remains poor due to a high rate of local recurrence and metastatic disease [3]. Treatment with standard chemotherapy offers a very modest prolongation of life with few, if any, cures [3]. There is desperate need to explore novel therapeutics to improve outcomes. The clinical need is so marked that Congress recently passed the *Recalcitrant Cancer Research Act* specifically to encourage further research into treatments for pancreatic cancer.

Erastin is a small molecule discovered during a screen of synthetic compounds with cytotoxic activity in cells containing mutations in the KRAS oncogene [4]. KRAS mutations are found in more than 90% of pancreatic cancers, and despite its frequency and being the earliest genetic alteration, previous attempts to selectively target KRAS have failed [5, 6]. Erastin has been shown to inhibit the cystine/glutamate antiporter (system x_c_^−^), resulting in the accumulation of reactive oxygen species (ROS) and non-apoptotic cancer cell death [7, 8]. System x_c_^−^ permits uptake of cystine, which is vital to the formation of cysteine, a key building block for the production of the antioxidant glutathione, an important contributor to cell homeostasis [9]. Additionally, Erastin has been reported to target mitochondrial voltage-dependent anion channels [10]. These channels are found in the outer mitochondrial membrane and facilitate transport of metabolites [11] and were reported to be important, but not sufficient, to result in Erastin-mediated cell death [10]. Erastin and its analogues have been explored in several clinical trials but have underperformed [12, 13]. We hypothesized that the lack of *in vivo* efficacy was caused by either a lack of targeted drug delivery and/or a failure of an efficient uptake mechanism by the cancer cells.

Targeting cytotoxic cancer therapeutics selectively to cancer cells represents a highly desired objective with potential to improve treatment efficacies and minimize off-target toxicities. Over the last decade, our laboratory has developed efficient means to selectively deliver small molecule drug cargoes into cancer cells based on the sigma-2 ligand/receptor concept [14]. We have demonstrated sigma-2 receptors to be overexpressed in most human malignancies, including pancreatic cancer [15]. Sigma-2 ligands have been shown to be rapidly internalized into cancer cells [16] and, at high doses, are capable of inducing apoptosis [15, 17-19]. Importantly, sigma-2 ligand conjugates retain the capacity to bind and deliver additional drug cargoes into cancer cells. The sigma-2 ligand portion provides targeting while the effector molecule provides functionality as part of the dual-domain therapeutic [14, 20, 21]. In summary, we have previously established proof-of-principal for sigma-2 ligand targeted cancer delivery with several small molecules, and this work represents the culmination of a highly collaborative multi-investigator effort focused on developing a new therapeutic with the goal of testing the sigma-2 cancer delivery concept clinically.

Herein, we report the synthesis and characterization of the novel small molecule drug conjugate SW V-49. This dual-domain compound was synthesized as a conjugate of the sigma-2 ligand SV119 with des-methyl Erastin (dm-Erastin). In these studies, we show that SW V-49 overcomes the cellular internalization block of dm-Erastin and reduces tumor sizes while improving survival in the best available models of pancreatic cancer. Our results suggest that SW V-49 is a highly potent pancreatic cancer therapeutic worthy of clinical investigation.

## RESULTS

### Synthesis of the sigma-2/dm-Erastin conjugate SW V-49

**Figure 1:**
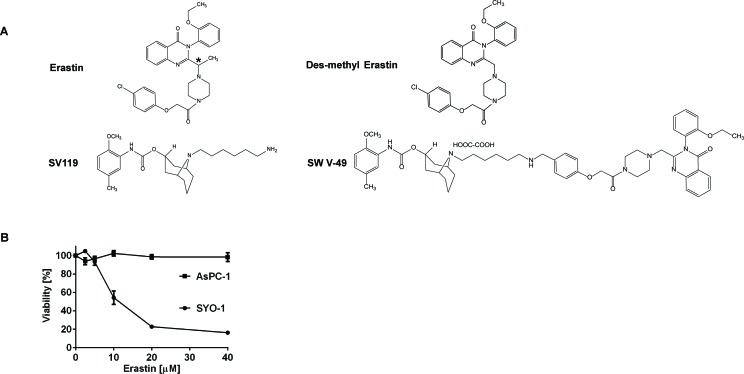
Chemical structures demonstrating the parental compounds and the novel drug conjugate SW V-49 **A.** The parental Erastin molecule (asterisk) and its des-methyl variant are nearly identical structurally. The sigma-2 ligand SV119 was chemically conjugated to dm-Erastin, resulting in the sigma-2/dm-Erastin conjugate SW V-49. **B.** SYO-1 synovial sarcoma cells were treated in a cell viability assay to demonstrate that Erastin is functionally active. In contrast, AsPC-1 pancreatic adenocarcinoma cells did not respond to Erastin, as demonstrated by lack of cellular death.

The main focus of our work was to develop novel therapies for evaluation in patients with pancreatic cancer. Erastin (Figure 1A) and its analogues are small molecules with cancer-selective cytotoxic activity profiles *in vitro* and thus represent attractive candidates for drug development [4]. The initial reports on Erastin analogues suggested that they might prove effective in pancreatic cancer patients, but they underperformed in clinical trials [12, 13]. We sought to understand why this drug class failed clinically and found that Erastin alone was incapable of inducing cell death in AsPC-1 and other pancreatic cancer cells *in vitro* while SYO-1 synovial cancer cells were sensitive (Figure 1B). We hypothesized that the lack of efficacy in pancreatic cancer might be caused by a deficiency in cellular drug uptake/internalization. Based on our previous experience utilizing sigma-2 ligands to deliver a payload to cancer cells [14, 20-22], we theorized that uptake blockade could potentially be overcome if we chemically conjugated Erastin to a sigma-2 ligand. We theorized that the conjugation would not sterically interfere with Erastin's function and cancer-selective lethality. An important criteria in choosing small molecules for further development into dual-domain drug conjugates is accessibility to chemical synthesis. The originally described Erastin molecule was challenging to synthesize due to an optically active carbon atom located within its structure (Figure 1A, asterisk). A slightly modified analogue, dm-Erastin, was reported to be functionally equivalent to the parental molecule as the methyl-substituted carbon is not necessary for its activity, thus avoiding the additional optically active structural site [23]. Thus, prior to generating the sigma-2/Erastin drug conjugate, we synthesized dm-Erastin (Figure 1A) and compared it with commercially available Erastin (EMD Millipore) in cell viability assays. Both drugs had identical cytotoxic activity profiles when assessed on Erastin-sensitive synovial sarcoma cells (data not shown). As a result of these comparisons, dm-Erastin was chemically linked to our sigma-2 delivery vehicle SV119 (Figure S1) resulting in the novel drug conjugate SW V-49 (Figure 1A).

### SW V-49 overcomes the chemotherapeutic plateau of dm-Erastin *via* enhanced drug uptake

**Figure 2:**
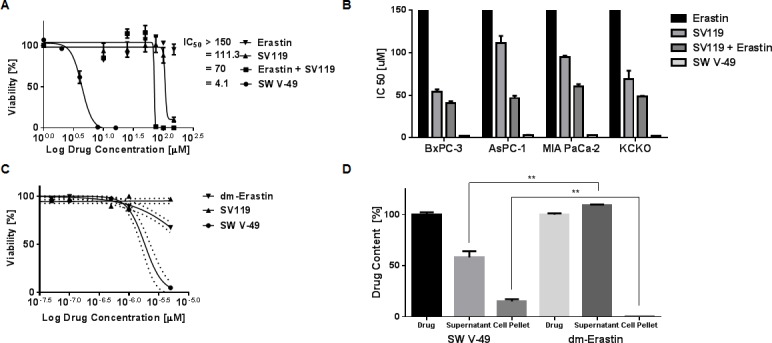
Conjugation to SV119 overcomes the internalization block of dm-Erastin and increases efficacy in pancreatic cancer cell lines **A.** PANC-1 cells were treated with increasing concentrations of SW V-49, SV119, Erastin, and an equimolar mix of SV119 and Erastin. CellTiter-Glo cell viability assay was performed 24 hours post-treatment. The half-maximal killing activities (IC_50_) of the various treatment conditions are expressed as means (*n* ≥ 3/group). **B.** The drug activity profiles (IC_50_ ± SEM) of SW V-49, Erastin, SV119, and the equimolar mix of SV119 and Erastin are shown across multiple pancreatic cancer cell lines (BxPC-3, AsPC-1, MIA PaCa-2, KCKO). SW V-49 treatment was effective and potent across all cell lines. **C.** Pancreatic cancer organoids (hM1 [41] is shown) were treated with increasing concentrations of SW V-49, SV119, and dm-Erastin. Cell viability was measured 72 hours after treatment using CellTiter-Glo. Only treatment with SW V-49 resulted in a robust cytotoxic response (IC_50_: 1.84 μM ± 1.10). Minimal effect of dm-Erastin and SV119 was observed. **D.** MIA PaCa-2 pancreatic adenocarcinoma cells were exposed to SW V-49 and dm-Erastin for 2 hours. Only SW V-49 was decreased from the media supernatant and present in the cell pellet, whereas dm-Erastin remained in the supernatant without any significant detection in the cell pellet.

As previously noted, all of our pancreatic cancer cell lines were resistant to Erastin. To test if conjugation to a sigma-2 ligand would overcome this treatment resistance, PANC-1 cells were treated with SW V-49, SV119 alone, dm-Erastin alone, and with equimolar mixtures of SV119 and dm-Erastin combined. Concentrations of dm-Erastin as high as 100 μM failed to demonstrate significant cytotoxic effects and its half-maximal killing concentration (IC_50_) was greater than 150 μM (Figure 2A). The moderate killing profile of the delivery agent SV119 was anticipated as reported previously [14, 17, 18]. Furthermore, equimolar mixes of dm-Erastin with SV119 did not lead to a substantial improvement over the baseline activity of SV119, as cytotoxicity was nearly identical to that of sigma-2 ligand alone (Figure 2A). In stark contrast to all other treatment groups, SW V-49 elicited a robust cytotoxic response with an IC_50_ in the single digit micromolar range. SW V-49 was 17-fold more effective than an equimolar mix of SV119 and dm-Erastin (Figure 2A, IC_50_: 4.1 μM ± 0.2 *vs* 70.0 μM ± 0.3, *p* < 0.05). This *in vitro* activity profile was confirmed across a panel of human- and murine-derived pancreatic cancer cell lines (Figure 2B).

Historically, clinical efficacy has not reliably followed success of novel therapies in long cultured cancer cell lines, and investigators have postulated that genetic drift and adaptation to cell culture conditions have contributed to a lack of translatability [24]. Patient-derived three dimensional cultures (organoids) are thought to better predict clinical successes [25, 26]. To assess the efficacy of SW V-49 in patient-derived organoid cultures, we treated multiple human pancreatic cancer organoid cultures (*n* = 5) with varying doses of SW V-49, SV119, and dm-Erastin (Figure 2C). As with the established cell lines, we observed a robust response following treatment with SW V-49, which resulted in an IC_50_ of 1.84 μM (SEM: ± 1.10). The controls behaved slightly different in organoids. The SV119 delivery molecule was nearly inactive and dm-Erastin exhibited a small cytotoxic response at the highest doses.

To test whether cellular uptake is improved *via* chemical conjugation with SV119, we utilized mass spectrometry and asked if pancreatic cancer cells were able to absorb SW V-49 from the culture supernatant when compared with non-conjugated dm-Erastin. Based on prior studies demonstrating rapid uptake of sigma-2 ligands into cancer cells [16], we treated human pancreatic cancer cells (MIA PaCa-2) with SW V-49 or dm-Erastin for two hours. The supernatants and cell pellets were then harvested and submitted for mass spectrometry. Drug-containing medium in the absence of cells was used to establish the baseline signal intensity for the respective compound. We found that dm-Erastin did not show any evidence for entering the cancer cells, also reflected by the fact that the drug concentration in the supernatant remained unaltered during the course of the experiment (Figure 2D). Extended exposure times and increased drug doses did not lead to accumulation of dm-Erastin in the cell pellets. In contrast, SW V-49 was found to be associated with the cell pellet, while the drug concentration in the supernatant fell concomitantly (Figure 2D). These data strongly support the hypothesis that the sigma-2 ligand delivery moiety of SW V-49 (SV119) is necessary and sufficient to facilitate efficient transport across the plasma membrane of pancreatic cancer cells, otherwise impermeable for the unconjugated drug cargo (dm-Erastin).

### SW V-49 reduces tumor growth and improves survival in syngeneic and patient-derived xenograft models of pancreatic cancer

**Figure 3:**
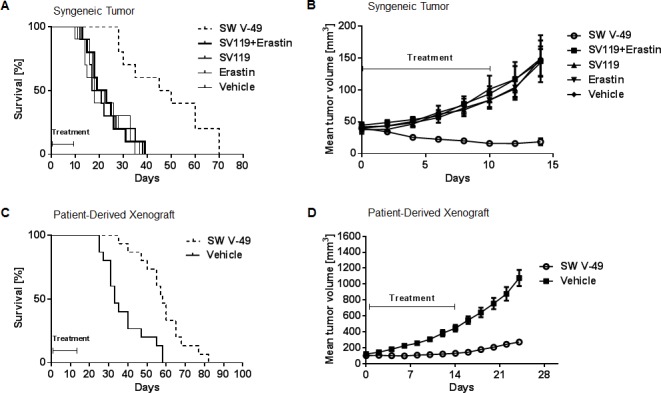
SW V-49 reduces tumor growth and improves median survival in murine models of pancreatic cancer **A.** C57BL/6 mice (*n* = 10 mice/group) with established KCKO tumors were treated daily i.p. with 375 nanomoles of the indicated compounds for 10 days. Kaplan-Meier survival curve of the mice is shown. Median survival of the group treated with SW V-49 was 48 days (SD: ± 16.9). All other groups had a significantly lower median survival (***p < 0.001). Mice treated with vehicle, Erastin only, SV 119 only, and an equimolar mix of SV119 and Erastin had median survivals of 20.0 ± 8.4 days, 18.0 ± 9.2 days, 19.0 ± 8.6 days, and 21.0 ± 7.9 days, respectively. **B.** There was no significant difference between DMSO, SV119, dm-Erastin, and the equimolar mix of SV119 and dm-Erastin (*p* = 0.9). Tumor volumes in mice treated with SW V-49 were significantly smaller compared in all control groups (***p < 0.001). **C.** Athymic nude mice (*n* = 15 mice/group) with established patient-derived xenograft tumors (PDX) were treated daily i.p. with 375 nanomoles of SW V-49 for 14 days. Mice treated with vehicle served as a control. A Kaplan-Meier survival curve of the mice is shown. Median survival of the group treated with SW V-49 was 58 days (SD: ± 12.5) compared to 33 days (SD: ± 11.5) for the control group (***p = 0.0002). **D.** Mice treated with SW V-49 demonstrated a dramatic difference in tumor volume compared to vehicle (****p < 0.0001).

To determine the *in vivo* efficacy of SW V-49, we employed several murine model systems of pancreatic cancer. We utilized genetically-engineered murine models driven by a KRAS mutation on a C57BL/6 background [27, 28] as well as a patient-derived xenograft model using athymic nude mice [29]. In the subcutaneous syngeneic model, C57BL/6 mice (*n* = 15 per group) were observed until tumors measured 5-6 mm in diameter and then treated with daily i.p. injections of 375 nanomoles of SW V-49, SV119, Erastin, an equimolar mix of SV119 and Erastin, or vehicle. SW V-49 more than doubled the median survival to 48 days (SD: ± 16.9); while vehicle (20.0 ± 8.4 days), Erastin (18.0 ± 9.2 days), SV119 (19.0 ± 8.6 days), and the equimolar mix of SV119 and Erastin (21.0 ± 7.9 days) all had significantly shorter survivals (Figure 3A, *p* < 0.0001). Only SW V-49 was capable of reducing the mean tumor volume during the treatment interval (Figure 3B, *p* = 0.0003). None of the other compounds, alone or in combination, resulted in a reduction of tumor size and all control groups exhibited similar growth rates when compared to vehicle alone (Figure 3B, *p* = 0.9).

Importantly, there were no treatment-related deaths or gross abnormalities in mouse behavior. In order to assess for more subtle toxicities, serum chemistries (AST, ALT, BUN, total protein, glucose, and Cr) and complete blood counts were analyzed and there were no significant differences (Tables S1 and S2). Necropsy, performed by a blinded veterinary pathologist employed by our Digestive Diseases Core facility, revealed no difference in mass (vehicle: 20.7± 0.6 grams; SW V-49: 21.3 ± 2.1 grams; *p* = 0.600) nor any injury to brain, heart, lungs, alimentary tract, kidneys, liver, or pancreas. Only mild peritonitis was identified at the site of repeated drug injections.

SW V-49 was evaluated in patient-derived xenograft models of pancreatic cancer, which our group and others believe complement the purely murine models [30-32]. Thirty mice bearing subcutaneous patient-derived tumors were randomized to receive daily treatment with i.p. injections of 375 nanomoles of SW V-49 or vehicle control for 2 weeks. SW V-49 nearly doubled median survival from 33 days (SD: ± 11.5) in the control group to 58 days (SD: ± 12.5) in the conjugate-treated group (Figure 3C, *p* = 0.0002). Additionally, SW V-49 markedly slowed the growth rate of the established tumors (Figure 3D, *p* < 0.0001). All mice tolerated the treatment well without obvious off-target effects.

We compared SW V-49 to a standard treatment for pancreatic cancer (gemcitabine) to understand the comparative magnitude of our drug's effect. In addition, we evaluated the effectiveness in yet another tumor model. The KP-2 tumor line, recently derived from a genetically-engineered spontaneously-arising model, was implanted orthotopically into C57BL/6 mice (*n* = 5-6 mice/group) to assess the efficacy of SW V-49 in a stroma-dense model (Figure S2). Similar genetic crosses have been demonstrated to recapitulate the tumor microenvironment from the stromal and immunologic perspectives to better model the human disease [33, 34].

**Figure 4:**
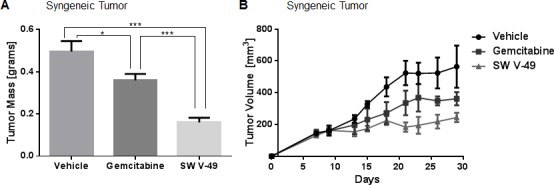
SW V-49 outperforms gemcitabine in stroma-dense models of pancreatic cancer **A.** C57BL/6 mice (*n* = 5-6 mice/group) with KP-2 orthotopic tumors were treated twice weekly with 20 mg/kg gemcitabine i.p. or with daily 200 nanomoles SW V-49 for two weeks once a palpable tumor had formed. Mice in the SW V-49 group had significantly smaller tumors compared to gemcitabine (***p = 0.0007) and vehicle (***p = 0.0003). **B.** C57BL/6 mice (*n* = 5 mice/group) with established KCKO tumors were treated twice weekly with 20 mg/kg gemcitabine i.p. or twice weekly with 200 nanomoles/injection SW V-49 i.p. for three weeks after tumors had grown to 5-6 mm in diameter. Mice in the SW V-49 group had significantly smaller tumors compared to gemcitabine (*p* = 0.049) and vehicle (*p* = 0.047).

Mice were randomized to receive i.p. injections twice weekly of 20 mg/kg gemcitabine or injections daily of 200 nanomoles SW V-49 for two weeks. Vehicle was utilized as a control. The dosing of SW V-49 was decreased to assess for potency with a smaller amount. Mice treated with SW V-49 had significantly smaller tumors compared to gemcitabine (*p* = 0.0007) and vehicle (*p* = 0.0003), indicating SW V-49 was effective in a stroma-dense model even at lower doses (Figure 4A). These data were repeated utilizing a genetically-engineered spontaneously-arising tumor line (KCKO) derived by another group [27, 28] *via* implanting tumors subcutaneously into C57BL/6 mice. When tumors reached 5-6 mm in diameter, mice were treated twice weekly with i.p. injections of 200 nanomoles SW V-49, 20 mg/kg gemcitabine, or vehicle alone. Mice treated with SW V-49 had significantly smaller tumors compared to both gemcitabine (*p* = 0.049) and vehicle groups (Figure 4B, *p* = 0.047).

In summary, SW V-49 effectively treated tumors in subcutaneous and orthotopic locations and results were reproducible in both patient-derived and genetically-engineered murine models. SW V-49 demonstrated efficacy without measurable toxicity and outperformed gemcitabine, a clinical standard for the treatment of pancreatic cancer.

### SW V-49 competes for the sigma-2 receptor with known sigma-2 binders and activates cargo-specific signaling pathways

**Figure 5:**
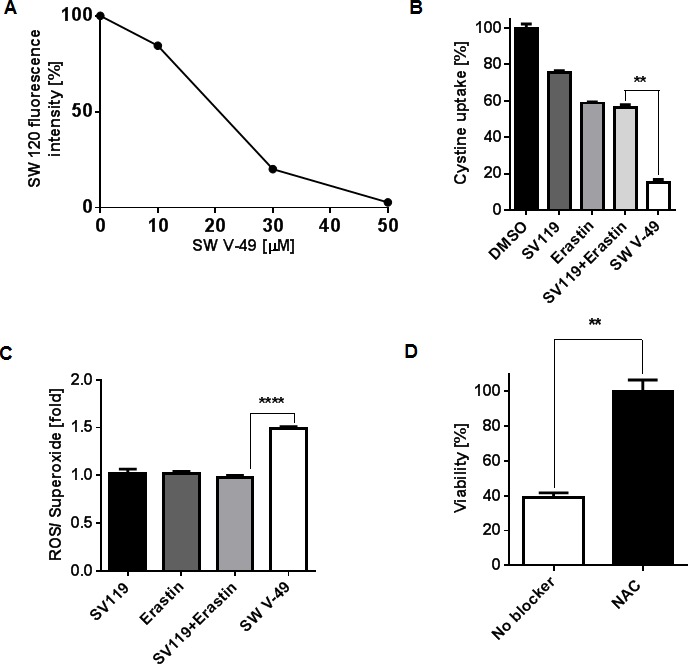
SW V-49 competes for the sigma-2 receptor and enacts Erastin-mediated death by inhibition of thesystem xc- cystine/glutamate antiporter leading to ROS production that is reversible with N-acetylcysteine rescue **A.** SW V-49 inhibits uptake of a fluorescently labeled sigma-2 ligand (SW120) in a dose-dependent manner. AsPC-1 cells were pretreated with increasing concentrations of SW V-49, followed by incubation with SW120 (10 nM), prior to analysis by flow cytometry. **B.** A cystine uptake assay was performed to assess the impact of the various compounds on the system x_c_^−^ antiporter. AsPC-1 cells were exposed for three minutes with SW V-49, SV119, Erastin, or an equimolar mix of SV119 and Erastin (200 μM). Uptake of ^14^C-labeled cystine was recorded by scintillography relative to DMSO treated control cells. Cystine uptake was reduced nearly 4-fold by 85% (SD: ± 2.7) in cells treated with SW V-49 compared to cells with an equimolar concentration of SV119 and Erastin (**p = 0.004). **C.** The production of ROS was measured as an indicator of effective Erastin-driven cytotoxic activity. AsPC-1 cells were treated with 8 μM of compound and ROS production was assayed one hour after treatment. Only SW V-49 treatment caused a ROS production level 1.5-fold (SD: ± 0.04 fold) higher than in all other treatment groups (****p < 0.0001). **D.** Addition of the antioxidant N-acetylcysteine (NAC) prevents SW V-49-mediated cell death (**p = 0.003). AsPC-1 cells were treated with NAC (10 mM) or vehicle and then exposed to SW V-49 (10 μM); NAC treatment rescued AsPC-1 cells from SW V-49.

SW V-49 is a new chemical entity, and it is highly unlikely that our conjugate should enter a cancer cell without utilizing the sigma-2 ligand/receptor complex. It would also be unexpected that SW V-49 should develop an entirely new mechanism of action. For completeness, we sought to confirm that SW V-49 binds to the sigma-2 receptor and retains Erastin's main mechanism of action. In order to demonstrate that SW V-49 binds to the sigma-2 receptor, we performed a competition assay with SW120, a fluorescently-labeled sigma-2 ligand [35], as previously described [21]. SW V-49 was able to block uptake of SW120 in a dose-dependent fashion (Figure 5A).

We further sought to verify that the mechanism of Erastin was preserved after conjugation to SV119. Erastin has been shown to initiate ROS-dependent cell death *via* blockade of the cystine/glutamate antiporter (system x_c_^−^) [7]. To elucidate the impact of our conjugate on the uptake of cystine, AsPC-1 cells were treated for three minutes with SV119, Erastin, an equimolar combination of SV119 and Erastin, and SW V-49. Cellular cystine uptake was monitored *via* scintillography using radiolabeled cystine. SV119 exhibited minimal impact on the uptake profile of radiolabeled cystine. Erastin alone had the anticipated effect, and the addition of SV119 did not enhance the blocking effect (Figure 5B). However, chemical conjugation of the two components, SW V-49, resulted in a marked decrease in cystine internalization by the cancer cells. There was an 85% (SD: ± 2.7) blockade with the conjugate compared to untreated cells. Compared to the mix of SV119 and Erastin (43 ± 2.5% blockade), we observed a 4-fold uptake reduction with the conjugate (Figure 5B, *p* = 0.004). We then assessed for ROS production to further confirm that the expected effector pathway of dm-Erastin was still functional with our conjugate. The strong effect of SW V-49 on the cystine/glutamate antiporter correlated with ROS production. After AsPC-1 cells were treated, the production of intracellular ROS increased significantly only for SW V-49 (Figure 5C, *p* < 0.0001). All other compounds, in isolation and as equimolar mixtures, did not result in elevated ROS levels and were similar to DMSO-treated control cells (Figure 5C). Cell death was completely abolished in the presence of the antioxidant N-acetyl cysteine (NAC) (Figure 5D, *p* = 0.003), further suggesting the ROS pathway is a major contributor of cancer cell death induced by SW V-49.

## DISCUSSION

Sigma-2 ligands have been shown to preferentially bind cancer cells [36], and we have previously described their capacity not only to image pancreatic cancers in animal models [15] but, even more importantly, in clinical imaging studies [37]. We and others have tested the safety of sigma-2 ligands and discovered that several of these compounds had intrinsic anti-cancer properties at high doses [17-19]. However, our observation that sigma-2 ligands were capable of internalizing additional drug cargoes provided the impetus for the development of more effective cancer-selective therapeutics [14, 20]. Previously, we tested delivery of peptides and chemotherapeutic agents and found that some small molecules retain their mechanism of action and efficacy when conjugated to select sigma-2 ligands. In several instances, conjugation to a sigma-2 ligand also enhanced delivery and internalization of the drug cargo [20, 21].

Erastin exhibits features that make it an attractive cargo for sigma-2 ligand-mediated cancer cell delivery. Erastin was initially described as a potent cytotoxic drug against RAS mutant cancers [10], and as KRAS mutations are found in more than 90% of pancreatic cancer patients [38], we hypothesized that an Erastin-based therapy would work effectively if we could overcome the uptake blockade observed in pancreatic cancer cells. We found our conjugate to be effective in KRAS-mutant and KRAS-wildtype pancreatic cancer cells. Subsequently, others have also found that the main mechanism of action for Erastin and its analogues is a non-apoptotic, oxidative form of cell death [7, 8]. We believe that our conjugate is so effective at generating ROS in cancer cells that it is impossible for us to differentiate subtle differences that might exist between KRAS-mutant and KRAS-wildtype cancers.

Through chemical conjugation, we successfully converted an Erastin analogue into a potent, targeted therapeutic (SW V-49) for pancreatic cancer with minimal off-site toxicity. Conjugation of dm-Erastin with SV119 overcame the internalization block that was observed in pancreatic cancer while preserving Erastin's inherent anti-tumor activity. We successfully demonstrated cellular association and reduction from the extracellular environment (supernatant) of SW V-49, while dm-Erastin remained in the media, despite the presence of pancreatic cancer cells in the culture vessels. We demonstrated that SW V-49 competed for the sigma-2 receptor with another well-established sigma-2 ligand suggesting the delivery and internalization function was preserved in the conjugate. SW V-49 also proved to be highly effective in three-dimensional pancreatic cancer organoid cultures. Success in this experimental *in vitro* setting may better predict drug efficacy at clinically achievable drug concentrations due to the ability of the organoid cultures to reproduce relevant aspects of disease progression unachievable in standard *in vitro* cell culture experiments.

The most promising finding of our current study was the ability of SW V-49 to effectively target pancreatic cancer in multiple murine models with minimal toxicity. We demonstrated the strong therapeutic response in several immunocompetent syngeneic models, a patient-derived xenograft model, and stroma-dense orthotopic models. Each of these models has features which recapitulate an aspect of the human disease and, taken together, we interpret success in all these models as a suggestion that SW V-49 has potential as a therapeutic for human pancreatic cancer. Only a short treatment interval with SW V-49 was required to reduce tumor sizes, delay tumor growth, and improve survival of our test animals. In addition, we found SW V-49 to be more effective in decreasing tumor burden than gemcitabine, a standard chemotherapeutic used clinically for pancreatic cancer. The cancer-selective delivery of SW V-49 may limit off-site toxicity and this supposition is supported by normal complete blood counts and serum chemistries in treated mice as well as by pathologic examination at necropsy.

Finally, we demonstrated preserved function of the Erastin analogue in inhibiting the system x_c_^−^ antiporter [7]. Pancreatic cancer cells have been reported to upregulate expression of system x_c_^−^[39], which leads to an enhanced capacity to import cystine, resulting in increased glutathione production. Here, we overcame the internalization block in pancreatic cancer and demonstrated a nearly 4-fold higher efficiency of SW V-49 than Erastin in inhibiting cystine uptake. This inhibition directly correlated with a robust increase in ROS production as quickly as one hour post-treatment and could be rescued by the antioxidant N-acetyl cysteine.

In summary, pancreatic cancer is a devastating malignancy and novel treatment approaches are desperately needed. Targeted delivery and efficient internalization of small molecules to pancreatic cancer cells represents one of the key advantages of the sigma-2 ligand-based drug concept. Tumor-selective delivery of the novel Erastin conjugate SW V-49 decreased tumor burden and increased survival while limiting off-site toxicities in preclinical mouse models of pancreatic cancer. Given the impressive findings using the best available models of pancreatic cancer explored in our current study, we believe further experimentation is highly warranted to rapidly advance this conjugate towards clinical evaluation.

## MATERIALS AND METHODS

### Small molecules

The originally identified Erastin molecule [10] was purchased from EMD Millipore (Billerica, MA). Sigma-2 ligand SV119 and Erastin analogue (dm-Erastin) were synthesized according to published methods [23, 40]. Synthesis of the sigma-2/dm-Erastin conjugate (SW V-49) is described in detail (Figure S1).

### Cell lines

The pancreatic cancer lines AsPC-1, BxPC-3, Mia PaCa-2, and PANC-1 were obtained from the American Type Culture Collection (ATCC, Manassas, VA). The synovial sarcoma cell line SYO-1 was provided by Dr. Van Tine. The mouse KCKO cell line isolated from a spontaneously developing pancreatic cancer overexpressing human MUC1 [27, 28] was kindly provided by Dr. Pinku Mukherjee (University of North Carolina, Charlotte, NC). The mouse KP-2 line was derived from pancreatic cancer tumor tissue obtained from p48-CRE/LSL-Kras^G12D^/p53^flox/+^ mice (backcrossed C57BL/6, *n* = 6). AsPC-1 and BxPC-3 cells were cultured in RPMI-1940 medium with 10% fetal bovine serum (FBS). MIA PaCa-2 cells were cultured in Dulbecco's Modified Eagle's Medium (DMEM) with 10% FBS and 2.5% horse serum. PANC-1 and SYO-1 cells were cultured in DMEM with 10% FBS. KCKO cells were cultured in RPMI-1940 medium with 10% FBS, 1% sodium pyruvate, 1% HEPES buffer, and 1% L-glutamine. KP-2 cells were cultured in 1:1 mixture of DMEM and Ham's F-12 Nutrient Mixture with 10% FBS. Penicillin (100 mg/mL) and streptomycin (100 mg/mL) were added to all media; cells were maintained in a humidified incubator at 37°C with 5% CO_2_. Cells were authenticated (every 6 months, last in October 2015) by morphology, doubling times, short tandem repeat profiling and tested for mycoplasma.

### Efficacy studies of the targeted drug conjugate SW V-49 *in vitro*

Cancer cell lines (pancreatic or sarcoma) were plated at a density of 2 × 10^4^ cells/well in black clear-bottom 96-well plates for 24 hours prior to treatment. The cells were treated for 24 hours with their respective drugs (alone, in combination, or as a conjugate). Untreated cells served as a control. Cytotoxicity was evaluated employing a CellTiter-Glo Luminescent Cell Viability Assay (Promega, Madison, WI) [20]. Luminescence signals were recorded using a multi-mode microplate reader (BioTek instruments, Winooski, VT). Different drug concentrations were assayed in triplicates.

### Human pancreatic tumor organoid culture

Organoids were isolated and grown as previously described [41] and dissociated into single cells using TrypIE (Life Technologies). For each well of a 384-well plate, 500 cells were plated in 50 μL mixture of human complete organoid media [41] supplemented with 10% GFR-Matrigel (Corning) and 10 μM Rho Kinase inhibitor Y-27632 (Sigma). Twenty-four hours after plating, 50 μL human complete organoid media containing SW V-49, SV119, or dm-Erastin was dispensed. Cell viability was measured 72 hours post-treatment using CellTiter-Glo Luminescent Cell Viability Assay. Experiment was replicated with cells from 5 different patients.

### Mass spectrometry detection of SW V-49 and dm-Erastin *in vitro*

MIA PaCa-2 cells were plated at a density of 5 × 10^5^ cells/well in clear-bottom 6-well plates for 24 hours prior to treatment. The cells were exposed to 8 μM SW V-49 or dm-Erastin. After 2 hours, the supernatant was aspirated. The remaining cells were washed twice with phosphate buffered saline (PBS) and harvested with trypsin/EDTA buffer (Life Technologies, Grand Island, NY). Empty wells were used to test drug stability in media at 37°C. Samples were analyzed by mass spectrometry at the Proteomics & Mass Spectrometry Facility at the Donald Danforth Plant Science Center. Cell samples were extracted using 1 mL 1:1 acetonitrile/methanol then dried down and resuspended to 100 μL. Media samples were diluted 30x prior to injection. Compounds were analyzed using a 4000 QTRAP equipped with a Shimadzu Prominence UPLC with an injection volume of 20 μL using standard gradient and solvents. Experimentation was performed in triplicates.

### *In vivo* assessment of SW V-49 in syngeneic and patient-derived xenograft models of pancreatic cancer

Animal studies were performed in adherence with the animal studies protocol approved by the Washington University Institutional Animal Care Facility. C57BL/6 mice (6 weeks old, National Cancer Institute Laboratories) were injected in the right flank with 200 μL of a single-cell suspension of KCKO cells in RPMI medium (2.5 × 10^5^ cells per mouse). Treatment began when the mean tumor diameter was 5-6 mm. Mice received daily i.p. injections with 375 nanomoles SW V-49, SV119, dm-Erastin, or equimolar combination of SV119 and dm-Erastin in 100 μL vehicle or vehicle alone (control) for 10 days. Vehicle was a mixture of 25% Cremophor in H_2_O. Tumors were measured in two dimensions every other day with a digital caliper, and tumor volumes were calculated by the standard formula of Tumor Volume = Length x Width^2^ x 0.5. Several mice from each treatment cohort were assessed for pathologic evaluation (Digestive Diseases Research Core Center at our institution). Blood was collected for complete blood count (CBC) and biochemical analysis (AST, ALT, BUN, total protein, glucose and Cr). Organs were examined grossly and histologically.

Surgical pancreatic adenocarcinoma specimens (3 × 3 mm pieces) were implanted subcutaneously into the flanks of anesthetized NOD/SCID mice [29, 30]. Stable patient-derived xenograft (PDX) lines were obtained after passaging the tumors three times. These tumors were implanted subcutaneously into the right flanks of athymic female nude mice (6 weeks old, National Cancer Institute Laboratories), and treatment began when tumors reached 5-6 mm in diameter. Mice were treated daily with i.p. injections of 375 nanomoles SW V-49 or control for 14 days. For all survival experiments, mice were euthanized and considered dead when the tumors reached a diameter of 2 cm or had ulcerated.

Syngeneic orthotopic pancreatic cancer tumors were established by surgical implantation. C57BL/6 mice (8 weeks old, Jackson Laboratory) were injected in the tail of the pancreas with 50 μL single-cell suspension of KP-2 cells (2.5 × 10^5^ cells per mouse) in Matrigel (Corning). On day 7 gross palpation revealed a tumor and mice were treated twice weekly with i.p. injections of 20 mg/kg gemcitabine (Tocris) or daily with 200 nanomoles SW V-49 for two weeks. Vehicle was used as a control. C57BL/6 mice (8 weeks old, Jackson Laboratory) were injected in the right flank with 100 μL single-cell suspension of KCKO cells (2.5 × 10^5^ cells per mouse) in PBS to create a syngeneic subcutaneous model. Treatment began when the tumors reached 5-6 mm in diameter. Mice were treated twice weekly with i.p. injections of 20 mg/kg gemcitabine (Tocris) or 200 nanomoles SW V-49 for three weeks. Vehicle was used as control.

### SW V-49 uptake studies by pancreatic cancer cells

AsPC-1 cells (5 × 10^5^/well) were seeded into 6-well plates 24 hours before treatment. The cells were incubated for 30 minutes at 37°C with 0 (control), 10, 30 and 50 μM of SW V-49 followed by addition of the fluorescently labeled sigma-2 ligand SW120 (10 nM). Thirty minutes after SW120 addition, the cells were washed twice with PBS and harvested with trypsin/EDTA buffer. The cells were washed twice with PBS, and SW120 internalization blockade by SW V-49 was determined by flow cytometry (FACSCalibur, BD Biosciences, San Jose, CA).

### Cystine uptake assay

Cystine uptake assay was performed as previously described [7]. Briefly, 5 × 10^5^ AsPC-1 cells/well were seeded overnight in a 6-well plate. Cells were washed twice in pre-warmed Na^+^-free uptake buffer (137 mM choline chloride, 3 mM KCl, 1 mM CaCl_2_, 1 mM MgCl_2_, 5 mM D-glucose, 0.7 mM K_2_HPO_4_, and 10 mM HEPES [pH 7.^4^]). Cells were incubated for 10 minutes at 37°C in 1 mL of uptake buffer to deplete cellular amino acids. This buffer was replaced with 600 μL uptake buffer containing 200 μM of compound (SW V-49, SV119, dm-Erastin, or an equimolar mix of SV119 and dm-Erastin) and 0.12 μCi (80 - 110 mCi/mmol) of L-[3,3′-^14^C]-cystine (American Radiolabeled Chemicals, St. Louis, MO) and incubated for 3 minutes at 37°C. DMSO was used as a control. Cells were washed three times with ice-cold uptake buffer and lysed in 500 μL of 0.1 M NaOH. To this lysate, 1 mL of scintillation fluid was added and radioactive counts per minute were obtained using a scintillation counter (Beckman instruments, Fullerton, CA.). Experiment was performed in triplicates.

### Detection of reactive oxygen species (ROS)

ROS measurement was performed using Total ROS/Superoxide Detection Kit (Enzo Life Sciences, Farmingdale, NY) according to the manufacturer's instructions. Briefly, AsPC-1 cells were seeded at a density of 2 × 10^4^ cells/well in black clear-bottom 96-well plates for 24 hours prior to treatment. Cells were treated with 8 μM SV119, Erastin, SW V-49, or an equimolar concentration of SV119 and Erastin. DMSO was used as a control. One hour post-treatment, culture supernatants were removed and replaced with 100 μL/well of ROS/Superoxide Detection Mix reagent. Fluorescent signal was measured using a multi-mode microplate reader (Bio-Tek). The assay was performed in 6 replicates.

### Inhibition of SW V-49-mediated ROS production with N-acetyl cysteine

AsPC-1 cells were plated at a density of 2 × 10^4^ cells/well in opaque 96-well, clear-bottom plates 24 hours prior to treatment. Cells were treated with N-acetyl cysteine (10 mM) or vehicle and exposed to SW V-49 (10 μM) for 5 hours. CellTiter-Glo Luminescent Cell Viability Assay was performed. Assay was performed in triplicate.

### Statistical analyses

Statistical analyses and data plotting were performed using GraphPad Prism software version 6.03 (San Diego, CA) in consultation with JL (biostatistician). Results were expressed as mean ± standard error of the mean of at least 3 biological replicates. IC_50_ values were calculated by curve fitting normalized viability *versus* drug concentration. One-way ANOVA was used to analyze the differences in IC_50_ values and SW V-49 inhibition with NAC tests. Student's two-tailed unpaired *t*-tests were used to evaluate the difference in SW V-49 tissue uptake, cystine uptake, ROS production, and tumor volume. Mann-Whitney test was used to compare the difference in CBC and biochemistry analyses. Kaplan-Meier survival analyses were used to assess differences between treatment groups and were compared using a log-rank test. Survival is reported as median survival ± standard deviation. Repeated measures ANOVA was used for analysis of tumor size. A *p-*value < 0.05 was considered significant for all analyses.

## SUPPLEMENTARY MATERIAL FIGURES AND TABLES



## Abbreviations

AST: aspartate aminotransferase
ALT: alanine aminotransferase
BUN: blood urea nitrogen
Cr: Creatinine
i.p: intraperitoneal
IC50: half maximal inhibitory concentration
