# Supplementary material for: Conjugation to the sigma-2 ligand SV119 overcomes uptake blockade and converts dm-Erastin into a potent pancreatic cancer therapeutic
Source: Oncotarget. 2016 May 22;7(23):33529–41. doi: 10.18632/oncotarget.9551 (PMC5085100; doi:10.18632/oncotarget.9551)
Supplement: Supplementary file 1 [file oncotarget-07-33529-s001.pdf]

# Conjugation to the sigma-2 ligand SV119 overcomes uptake blockade and converts dm-Erastin into a potent pancreatic cancer therapeutic

**Supplementary Material**

**Synthesis of SW V-49**

Chemical synthesis of the sigma-2 ligand **SV119** was performed as previously described [40]. The synthesis of **SW V-49s** was outlined in scheme 1.

$^1$H NMR spectra were recorded on a Varian 400 MHz NMR spectrometer. Chemical shifts are reported in δ values (parts per million, ppm) relative to an internal standard of tetramethylsilane (TMS). The following abbreviations are used for multiplicity of NMR signals: br s = broad singlet, d = doublet, m = multiplet, q = quartet, s = singlet, t = triplet. Melting points were determined on an Electrothermal melting point apparatus and are uncorrected. Elemental analyses were performed by Atlantic Microlab, Inc., Norcross, GA and were within ± 0.4% of the calculated values. All reactions were carried out under an inert atmosphere of nitrogen.

**Ethyl 2-(4-formylphenoxy)acetate (1).**

Ethyl 2-bromoacetate (3.7 g, 22.0 mmol)) and potassium carbonate (8.3 g, 60.0 mmol) were added into the solution of 4-hydroxybenzaldehyde (2.4 g, 20.0 mmol) in acetonitrile (60 mL). The reaction mixture was refluxed for 24 h. After cooling, the reaction mixture was filtered, and evaporated to give **1** as a light yellow liquid (4.1 g, quantitative). $^1$H NMR ($CDCl_3$) δ 9.90 (s, 1H), 7.85 (d, *J* = 8.4 Hz, 2H), 7.01 (d, *J* = 8.4 Hz, 2H), 4.71 (s, 2H), 4.28 (q, *J* = 7.1 Hz, 2H), 1.31 (t, *J* = 7.1 Hz, 3H).

**2-(4-Formylphenoxy)acetic acid (2).**

Hydrolysis of **1** (4.1 g, 20.0 mmol) with sodium hydroxide (2.2 eq) in methanol/water (2:1, 90 mL) for 24 h, followed by acidifying with 10% HCl solution gave **2** as an off-white solid (3.1 g, 87% yield). $^1$H NMR (DMSO-$d_6$) δ 13.18 (br s, 1H), 9.91 (s, 1H), 7.89 (d, *J* = 8.6 Hz, 2H), 7.13 (d, *J* = 8.6 Hz, 2H), 4.86 (s, 2H).

**2-(2-Chloroacetamido)benzoic acid (3).**

Triethylamine (4.06 g, 40.1 mmol) was added in the solution of 2-aminobenzoic acid (5.0 g, 36.5 mmol) in dichloromethane (90 mL) and the mixture was cooled in an ice-water bath. A solution of chloroacetyl chloride (4.5 g, 40.1 mmol) in dichloromethane (40 mL) was added dropwise and the mixture was allowed to stir at ambient temperature overnight. The solid was filtered and washed with cold water followed by 5% diethyl ether in hexane and was air dried to afford compound **3** as a white solid (7.4 g, 95% yield). $^1$H NMR (DMSO-$d_6$) δ 11.82 (s, 1H), 8.52 (d, *J* = 7.5 Hz, 1H), 8.01 (d, *J* = 7.5 Hz, 1H), 7.63 (t, *J* = 7.5 Hz, 1H), 7.21 (t, *J* = 7.5 Hz, 1H), 4.45 (s, 2H), 3.35 (br s, 1H).

**2-(Chloromethyl)-3-(2-ethoxyphenyl)quinazolin-4(3H)-one (4).**

Phosphoryl chloride (6.5 g, 42.4 mmol) was added dropwise to a mixture of **3** (3.1 g, 14.5 mmol) and 2-ethoxyaniline (2.0 g, 14.5 mmol) in acetonitrile (50 mL). The mixture was heated at reflux overnight. The reaction mixture was cooled to room temperature, poured into a slurry of ice/saturated solution of $Na_2CO_3$. The resulting solid was filtered, washed with water and air dried to give **4** as a brown solid (2.5 g, 53% yield). $^1$H NMR ($CDCl_3$) δ 8.31 (d, *J* = 7.8 Hz, 1H), 7.78-7.82 (m, 2H), 7.47-7.55 (m, 2H), 7.35 (d, *J* = 7.4 Hz, 1H), 7.06-7.14 (m, 2H), 4.35 (d, *J* = 11.9 Hz, 1H), 4.17 (d, *J* = 11.9 Hz, 1H), 4.06 (q, *J* = 6.9 Hz, 2H), 1.23 (t, *J* = 6.9 Hz, 3H).

**3-(2-Ethoxyphenyl)-2-(piperazin-1-yl-methyl)quinazolin-4(3H)-one (5).**

Piperazine (2.7 g, 32.0 mmol) was added to a mixture of **4** (2.5 g, 8.0 mmol), $K_2CO_3$ (4.4 g, 32.0 mmol) and KI (1.7 g, 10.4 mmol) in acetonitrile (75 mL). The reaction mixture was heated at 85-90 $^0$C overnight. After cooling, it was filtered and the solid was washed with acetonitrile. The combined organic layers were evaporated. The resulting residue was dispersed in water and extracted with ethyl acetate. The organic layers were washed with brine, dried over $Na_2SO_4$, filtered and evaporated. The crude residue was purified by column chromatography (10% methanol, 0.5% $NH_4OH$ in dichloromethane) to give **5** as a yellow oil (2.3 g, 79% yield). $^1$H NMR ($CDCl_3$) δ 8.30 (d, *J* = 7.8 Hz, 1H), 7.75-7.77 (m, 2H), 7.39-7.50 (m, 2H), 7.28 (d, *J* = 7.0 Hz, 1H), 7.01-7.08 (m, 2H), 4.05 (q, *J* = 6.8 Hz, 2H), 3.18-3.28 (m, 2H), 2.73 (s, 4H), 2.33-2.37 (m, 2H), 2.17-2.20 (m, 2H), 1.51 (br s, 1H), 1.22 (t, *J* = 6.8 Hz, 3H).

**4-(2-(4-((3-(2-Ethoxyphenyl)-4-oxo-3,4-dihydroquinazolin-2-yl)methyl)piperazin-1-yl)-2-oxoethoxy)benzaldehyde (6).**

A solution of DCC (0.500 g, 2.4 mmol) in acetonitrile (4 mL) was added to a cooled mixture of acid **2** (0.360 g, 2.0 mmol), *N*-hydroxysuccinimide (0.280 g, 2.4 mmol) in acetonitrile (12 mL). After stirring at room temperature for 45 min, a solution of **5** (0.800 g, 2.2 mmol) in acetonitrile (10 mL) was added, then continued stirring overnight. The solid was filtered off and the filtrate was evaporated. The resulting residue was purified by column chromatography (8% methanol in dichloromethane) to give **6** as an off-white solid (0.847 g, 80% yield). $^1$H NMR ($CDCl_3$) δ 9.89 (s, 1H), 8.27-8.31 (m, 1H), 7.72-7.84 (m, 4H), 7.42-7.52 (m, 2H), 7.25 (d, *J* = 6.3 Hz, 1H), 7.02-7.09 (m, 4H), 4.74 (s, 2H), 4.05 (q, *J* = 7.0 Hz, 2H), 3.42-3.49 (m, 4H), 3.22-3.31 (m, 2H), 2.18-2.46 (m, 4H), 1.22 (t, *J* = 7.0 Hz, 3H).

**9-(6-((4-(2-(4-((3-(2-Ethoxyphenyl)-4-oxo-3,4-dihydroquinazolin-2-yl)methyl)piperazin-1-yl)-2-oxoethoxy)benzyl)amino)hexyl)-*N*-(9-azabicyclo[3.3.1]nonan-3α-yl)-*N*-(2-methoxy-5-methylphenyl)carbamate oxalate(SW V-49s).**

A solution of amine **SV119** (0.386 g, 0.95 mmol) in dichloromethane (4 mL) was added into a solution of **6** (0.480 g, 0.91 mmol) in dichloromethane (4 mL). The mixture was stirred for 4 h then the solvent was evaporated. The residue was dissolved in ethanol (5 mL), then $NaBH_4$ (0.100 g, 2.6 mmol) was added. The mixture was stirred for 6 h, then quenched with 10% HCl solution. After the solvent was evaporated, it was basified with 10% NaOH solution, extracted with dichloromethane and evaporated. The resulting residue was purified by column chromatography (10% methanol, 0.5% $NH_4OH$ in dichloromethane) to give the product as free amine (0.460 g, 55% yield). $^1$H NMR ($CDCl_3$) δ 8.30 (d, *J* = 7.8 Hz, 1H), 7.94 (s, 1H), 7.74-7.78 (m, 2H), 7.41-7.51 (m, 2H), 7.23-7.26 (m, 3H), 7.14 (s, 1H), 7.03-7.09 (m, 2H), 6.86 (d, *J* = 8.6 Hz, 2H), 6.73-6.79 (m, 2H), 5.10-5.16 (m, 1H), 4.62 (s, 2H), 4.04 (q, *J* = 6.9 Hz, 2H), 3.85 (s, 3H), 3.74 (s, 2H), 3.43-3.48 (m, 4H), 3.22-3.30 (m, 2H), 3.12 (br s, 2H), 2.60-2.64 (m, 4H), 2.37-2.50 (m, 4H), 2.29 (s, 3H), 2.19-2.25 (m, 3H), 1.90-1.96 (m, 2H), 1.48-1.58 (m, 7H), 1.28-1.34 (m, 6H), 1.22 (t, *J* = 6.9 Hz, 3H). The oxalate salt was prepared using 1 equivalent of oxalic acid in ethanol to give **SW V-49s** as an off-white solid (470 mg), mp 164-165 $^0$C. Anal.

($C_{55}H_{69}N_7O_{11}\cdot 2H_2O$): Calculated, %: C 63.51; H 7.07; N 9.43, Found, %: C 63.54, H 7.06, N 9.76.

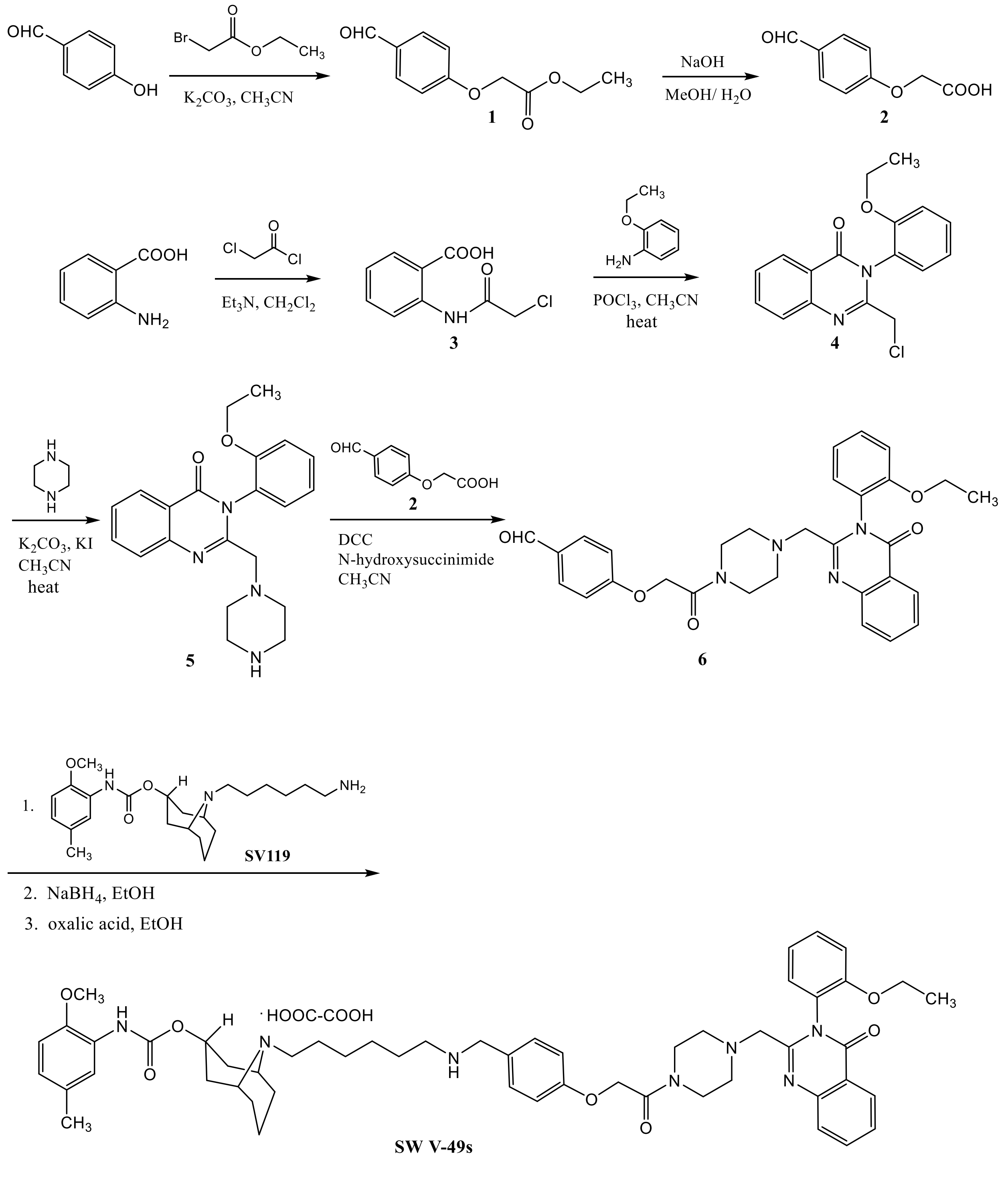


**Figure S1. Synthesis schematic of SW V-49**

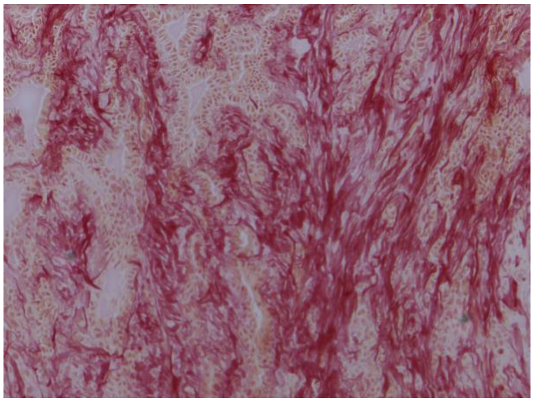

**Figure S2. KP-2 subcutaneous tumor demonstrating dense stroma.**

The KP-2 pancreatic tumor line, derived from a genetically-engineered spontaneously arising model, was assessed by immunohistochemistry; dense stroma is identified by Sirius Red staining.

**Table S1. SW V-49 does not induce changes in blood cytology (CBC) following treatment of tumor-bearing C57BL/6 mice.**

| | **Control** | **SW V-49** | ***p*-value** |
|---|---|---|---|
| **WBC ($10^3$/µL)** | 7.1 ± 1.4 | 4.4 ± 1.2 | 0.200 |
| **RBC ($10^6$/µL)** | 9.0 ± 0.2 | 8.2 ± 0.5 | 0.100 |
| **HGB (g/dL)** | 11.7 ± 0.6 | 10.3 ± 0.5 | 0.100 |
| **HCT (%)** | 46.5 ± 2.4 | 39.8 ± 2.4 | 0.100 |
| **MCV (fL)** | 51.8 ± 1.8 | 48.3 ± 2.7 | 0.200 |
| **MCH (pg)** | 13.0 ± 0.5 | 12.5 ± 0.3 | 0.200 |
| **MCHC (%)** | 25.2 ± 0.6 | 26.0 ± 1.2 | 0.400 |
| **Platelets ($10^3$/ µL)** | 728.0 ± 70.1 | 748.3 ± 137.0 | 0.900 |

Blood cytology analysis of C57BL/6 mice (n = 3 mice/group) treated with SW V-49 and vehicle (control) for 10 days. The differences in complete blood count laboratory values between the two groups are not statistically significant.

**Table S2. SW V-49 does not induce changes in serum chemistry following treatment of tumor-bearing C57BL/6 mice.**

| | **Control** | **SW V-49** | ***p*-value** |
|---|---|---|---|
| **BUN (mg/dL)** | 22.7 ± 5.5 | 19.3 ± 2.5 | 0.500 |
| **Creatinine (mg/dL)** | 0.29 ± 0.036 | 0.22 ± 0.015 | 0.100 |
| **ALT (μ/L)** | 222.0 ± 246.4 | 88.33 ± 43.0 | 0.400 |
| **AST (μ/L)** | 291.7 ± 366.7 | 123.0 ± 59.7 | 0.900 |
| **Total Protein (g/dL)** | 5.7 ± 0.4 | 5.3 ± 0.3 | 0.300 |
| **Glucose (mg/dL)** | 182.0 ± 4.4 | 222.7 ± 75.6 | 0.825 |

Biochemical analysis of C57BL/6 mice (n = 3 mice/group) treated with SW V-49 and vehicle (control) for 10 days. The differences in serum chemistries between the two groups are not statistically significant.
